# *FEF2575* percentage reversibility and its comparison with *FEV1 Reversibility* in diagnosis of asthma

**DOI:** 10.12669/pjms.41.9.11535

**Published:** 2025-09

**Authors:** Mirza Muhammad Ayub Baig, Muhammad Usman Baig, Mehr Muhammad Imran

**Affiliations:** 1 Mirza Muhammad Ayub Baig Department of Pulmonology, Services Hospital/SIMS, Lahore, Pakistan; 2 Muhammad Usman Baig Ameer-ud-Din Medical College, Lahore, Pakistan; 3 Mehr Muhammad Imran Department of Pulmonology, Services Hospital/SIMS, Lahore, Pakistan

**Keywords:** Bronchial asthma, Forced expiratory flow at 25% to 75% of forced vital capacity (FEF2575), Forced expiratory volume in one second (FEV1), Forced vital capacity (FVC), Peak Expiratory Flow (PEF)

## Abstract

**Background & Objective:**

Bronchial asthma is a chronic inflammatory disease with reversible airflow obstruction. Smaller airways are primary and most common site of inflammation in bronchial asthma. *FEF2575* is a measure of small airway function and may serve as a useful marker in the diagnosis of asthma. Conventional spirometry criteria, particularly bronchodilator reversibility of *FEV1*, exhibit limited sensitivity, potentially leading to missed diagnoses of asthma. There is a need for more parameters like *FEF2575* reversibility to enhance diagnostic accuracy within this population. Our objective weas to evaluate whether inclusion of *FEF2575* reversibility improves the diagnostic sensitivity of spirometry in patients clinically suspected of having asthma.

**Methodology:**

The study included patients who visited the SGRH pulmonary clinic between March to December, 2022. Patients suspected of asthma as per GINA criteria underwent spirometry with bronchodilator reversibility testing, followed by a one-month therapeutic trial. Only those with symptomatic improvement were classified as Clinically confirmed asthma cases and selected for final analysis. A total of 304 non-asthmatic individuals were included as a control group for comparison.

**Results:**

A total of 599 individuals, including 295 probable asthmatics, were included in the study. The mean age of participants was 43.2±17.8 years Clinically confirmed asthmatic patients exhibited significantly higher reversibility across all three parameters—*FEV1, FEF2575*, and FVC—compared to healthy individuals (p-value <0.05). The sensitivity of *FEV1, FEF2575*, and FVC was 0.31, 0.59, and 0.29, respectively, with a cut-off percentage reversibility of >12%.

**Conclusion:**

Incorporating *FEF2575* reversibility alongside standard parameters enhances diagnostic sensitivity and may aid in timely confirmation of asthma, potentially improving clinical outcomes through more accurate diagnosis.

## INTRODUCTION

Bronchial asthma is a common respiratory disease affecting 18% of the world’s population including adults and children.^1^,^2^ “Bronchial asthma is a chronic inflammatory disease characterized by airflow obstruction which is reversible suddenly either itself or after treatment.”^1^ In children, persistent bronchial asthma may lead to arrested growth of lungs and subsequently early decline of *FEV1* in adult age.^3^

Bronchial asthma if left untreated in adults, may lead to remodeling of airways due to persistent airway inflammation.^4^ As a consequence, the patients develop airway obstructive disease with partial reversibility, and in some cases, no reversibility of airways and respiratory failure.^5^

Accurate diagnosis of asthma and prompt treatment are necessary to prevent a decline in lung function.^6^ Bronchial asthma is suspected on symptoms and examination of the chest. Diagnosis of bronchial asthma is based on *FEV1* or FVC reversibility, which is >12% after inhalation with a rapid-acting bronchodilator like salbutamol or formoterol.^2^

Smaller airways are primary and most common site of inflammation in bronchial asthma.^7^ *FEF2575* is forced expiratory flow between 25% and 75% of forced vital capacity, generally considered as an approximate measure of the caliber of the distal airway. Its reduction represents a small airway obstruction caused by asthma inflammation. Therefore, impairment of *FEF2575* may be a marker of an early reduction of pulmonary function in asthma and may or may not be associated with *FEV1* reversibility.^8^

## METHODOLOGY

This was a prospective cross-sectional study. The study included patients of clinically confirmed bronchial asthma and healthy individuals who visited the SGRH pulmonary clinic between March to December, 2022. Patients were initially suspected of having asthma based on symptoms using the Criteria given by GINA 2022 guidelines and mentioned in operational definitions.^2^

### Ethical Approval:

The ethical review board of Sir Ganga Ram Hospital (SGRH)/Fatima Jinnah Medical University (FJMU) approved the study (No.18-Art/Pulmon-IRB/FJ; Dated: May 12, 2018). Written consent was taken from all the individuals before including them in the study.

All clinically suspected cases underwent spirometry to assess baseline pulmonary function parameters including *FEV1*, *FEF2575*, FVC. MIR minispir It was advised to perform three efforts pre and post bronchodilator and best effort was selected. Reversibility of these parameters was evaluated using spirometry 15 minutes after bronchodilator (400mcg salbutamol) inhalation via nebulizer. Subsequently, a one-month therapeutic trial with standard asthma treatment was conducted as part of the clinical diagnostic process.^2^ Only those patients who demonstrated clear symptomatic improvement following the treatment trial were considered to have clinically confirmed asthma and were included in the final analysis. Symptomatic improvement served as the primary criterion for clinical confirmation. Patients were initially suspected and clinically diagnosed with asthma based on the GINA 2022 guidelines. A control group comprising 304 non-asthmatic healthy individuals was included to demonstrate the absence of significant bronchodilator reversibility in individuals without asthma.

### Exclusion criteria:

Individuals were excluded if:

- They had diseases that may manifest by wheezing like congestive cardiac failure, foreign body in airways, and tracheal obstruction were excluded clinically.
- They were Smokers
- They had used short-acting bronchodilators within six hours or long-acting bronchodilator within 12 hours.
- They had a history of respiratory tract infection within four weeks were excluded as well.
- 57 patients who did not follow up were excluded from the study.

### Operational Definition:

### Healthy Individual:

Those individuals who have no history of shortness of breath, chest tightness, and wheezing with or without respiratory tract infection for the last twelve months.

### Suspected asthma:^2^

A score of >1 point was labeled as suspected asthma based on the following clinical scoring algorithm.

- Diurnal variation of symptoms (1 point).
- Recurrent episodes (2 points).
- medical history of allergic diseases (1 point).
- wheezing sound (2 points).

### Clinically confirmed asthma:^2^

Patients were labeled as Clinically confirmed Asthma if they showed improvement in symptoms after 4-week therapeutic trial with six micrograms of formoterol and 400 micrograms of budesonide inhaled therapy.

### Statistical analysis:

Statistical Package for Social Sciences (SPSS) version 27 was used for analysis. Categorical variables were expressed using frequency and percentages. Continuous variables were reported using means and standard deviation. Kolmogorov–Smirnov test was used to establish normality of continuous variables. Continuous variables were compared using two-sample T-test. Correlation between continuous and categorical variables was measured using Point-biserial correlation. Receiver Operating Characteristic (ROC) curves were constructed to evaluate the sensitivity and specificity of various spirometry parameters in diagnosing asthma.

**Fig.1:**
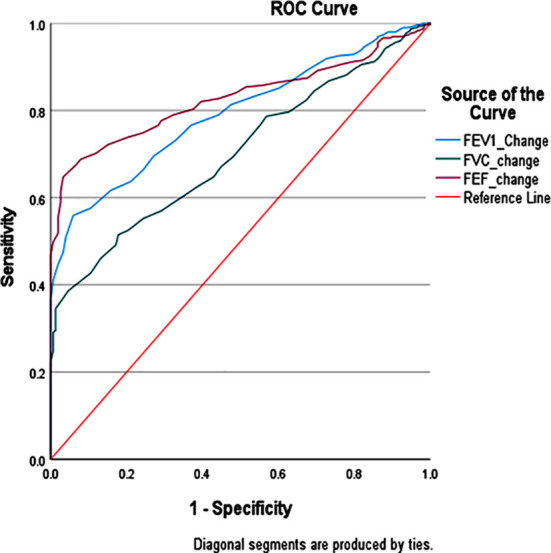
*ROC* curve of *FEV1, FEF2575* and *FVC %* reversibility.

## RESULTS

After exclusion, a total of 599 individuals were included in the study. There were slightly more males than females in the study. Mean age of all the individuals was 43.2±17.8 years. 295 individuals were clinically confirmed asthmatics according to GINA guidelines. 304 healthy individuals were included for comparison. The demographics are discussed in Table-I.

**Table-I:** Age, gender, and percentage predictive value of *FEV1, FEF2575,* and *FVC* before bronchodilator of asthmatic and non-asthmatic patients.

Variable		Asthmatic (n=295)		Non-Asthmatic (n=304)	
		Mean or frequency	SD	Mean or frequency	SD
Gender (Frequency)	Male	169 (57%)		166 (54%)	
	Female	126 (43%)		138 (46%)	
	Total	295		304	
Age (Mean)	Male	47.3	18.5	39.8	16.36
	Female	47.0	18.4	38.8	16.31
	Total	47.2	18.4	39.3	16.32
Percentage predictive value of FEV1 before bronchodilator	Male	76.3	25.5	95.3	18.0
	Female	73.46	19.8	95.2	15.5
	Total	75.1	23.2	93.9	16.9
Percentage predictive value of FEF2575 before bronchodilator	Male	76.4	33.4	106.8	34.1
	Female	74.4	25.9	94.9	23.9
	Total	75.6	30.4	101.4	30.4
Percentage predictive value of FVC before bronchodilator	Male	70.7	23.5	91.6	16.1
	Female	68.2	78.0	91.6	16.7
	Total	69.6	21.4	91.6	16.3

Clinically confirmed asthmatic patients had significantly higher reversibility of all three parameters i.e. *FEV1*, *FEF2575*, and FVC, as compared to the healthy individuals (p-value <0.05). The FEV1, *FEF2575*, and FVC% reversibility of all individuals were analyzed. Higher % reversibility of *FEV1*, *FEF2575*, and FVC was correlated with Asthma (R=0.498,0.537 and 0.376 respectively). At a cut-off value of >30%, the sensitivity of *FEF2575* reversibility was 0.20. The sensitivity and specificity of *FEV1*, PEF, and *FEF2575* were calculated and shown in Table-II.

**Table-II:** Sensitivity and specificity of different parameters for Asthma.

Parameter	Area under Curve	Cut of value	Sensitivity	Specificity
FEV1 %reversibility	0.792	>12%	0.305	1
		>15%	0.244	1
FEF2575 %reversibility	0.828	>12%	0.590	0.974
		>15%	0.498	1
FVC %reversibility	0.702	>12%	0.295	0.987
		>15%	0.247	0.993

The area under the curve of %reversibility of *FEV1*, *FEF2575*, and FVC was 0.792, 0.794 0.828, and 0.702 respectively. When using the standard diagnostic criterion of >12% reversibility along with ≥200 mL absolute improvement, *FEV1* and FVC together classified 112 clinically confirmed asthma patients as positive by spirometry criteria, yielding a sensitivity of 0.38. Of these, 89 met both criteria, while 23 met only one. Considering *FEF2575* reversibility (>12% only) along with standard criteria of *FEV1* and FVC increased the combined sensitivity of all three parameters to 0.75. Notably, *FEF2575* alone classified an additional 109 patients who did not meet the *FEV1* or FVC criteria. 74 patients still remained unclassified.

## DISCUSSION

In this study, 61.4% of patients demonstrated a >12% reversibility in *FEF2575* following bronchodilator administration. This was considerably higher than the proportion of patients who met conventional spirometric criteria, with only 30.5% showing >12% reversibility in *FEV1* and 29.5% in FVC. Notably, *FEF2575* alone classified an additional 109 patients who did not meet the *FEV1* or FVC criteria, highlighting its potential utility in detecting small airway dysfunction that may otherwise go unrecognized.

Fillard et al. has reported a 30.4% sensitivity for flow-related bronchodilator reversibility of *FEV1*, defined as an improvement of ≥12% from baseline.^9^ *Janson et al*. found a lower sensitivity of 17%, using a threshold of 200 mL improvement in *FEV1* after inhalation of 200 mcg of salbutamol.^10^ In another study, *DJ et al*. found *FEV1* reversibility sensitivity to be 22% in the adult population of the Tasmanian island based on a longitudinal study for diagnosis of asthma.^11^ In our study, we found it 30.5% at >12% *FEV1* reversibly which is higher than the above-mentioned studies.

Another important value is *FEF2575* of the flow volume loop which represents the forced expiratory airflow in distal or smaller airways. Some studies suggest that smaller airways are more commonly affected by the inflammation in moderate to severe bronchial asthma as compared to larger airways.^7^,^8^ *DR et al*. analyze the usefulness of *FEV1* and *FEF2575* bronchodilator reversibility in the diagnosis and severity of bronchial asthma in children.^12^ They defined bronchodilator responsiveness as a ≥12% improvement in *FEV1* and a ≥30% improvement in *FEF2575*. Based on this criterion, they observed *FEF2575* reversibility in 16% of cases, which is significantly lower than the 59% sensitivity observed in our study when using a >12% reversibility cutoff for *FEF2575*. In our analysis, we specifically assessed *FEF2575* reversibility at the ≥30% threshold as well and found the sensitivity to be 20% for detecting asthma. Although they consider 30% reversibility of *FEF2575* to label patients as bronchodilator responsive which is higher than our criteria, there is no international recommendation for this value.^12^ *Philips H et al*. evaluated the efficacy of *FEF2575* and FEF75 in detecting airway obstruction. They found *FEF2575* is effective in detecting airway obstruction but they did not proceed for its benefit in reversibility measurement, giving the impression of no clinical significance.^13^

**Fig.2:**
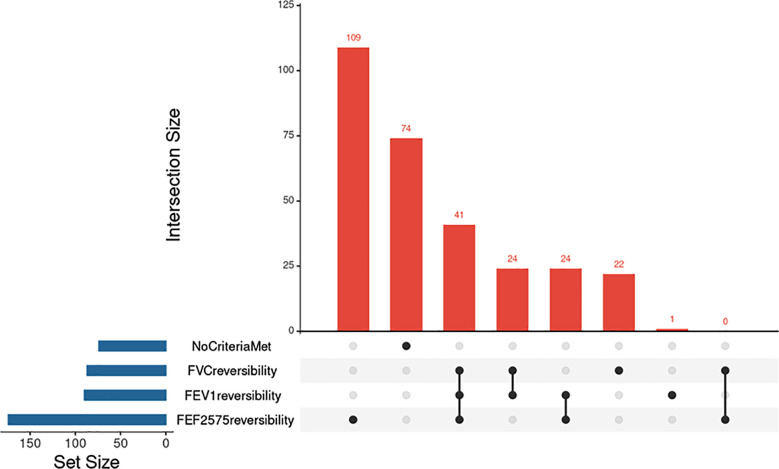
Up Set plot illustrating the overlap in patients showing reversibility in FEV1, FVC, and FEF2575 after bronchodilator use. A cutoff value of >12% reversibility was used for all parameters.

Bronchial asthma is a chronic inflammatory disease which can involves small airways and large airways. In recent studies, it was elaborated that small airways are the main and most common site of inflammation in bronchial asthma.^7^ Current criteria for diagnosis of bronchial asthma is based on reversibility of *FEV1* >12% or 200ml with a bronchodilator.^2^ *FEV1* primarily reflects airflow in the proximal large airways and is less sensitive to small airway obstruction.^14^ There are various studies which shows *FEF2575* can predict small airways disease in bronchial asthma.^15^ Therefore, we propose that *FEF2575* reversibility should be added to in criteria for diagnosis of bronchial asthma.

Future research should focus on establishing standard cutoff values for *FEF2575*% reversibility, as current guidelines lack consensus on its diagnostic thresholds in asthma. Additionally, more studies with larger sample size in different group of populations are needed to validate these findings and enhance their applicability in these groups. Longer follow-up periods should also be incorporated to assess the long-term diagnostic accuracy particularly in patients who do not meet traditional *FEV1* or FVC reversibility criteria.

### Limitations:

Spirometry was not repeated after one month of follow-up due to limited resources and patients were assessed clinically only. This may have affected the objectivity of outcome evaluation. It was a single-center study with a relatively small sample size, which may limit its applicability to other group of population and statistical power. Additionally, patients were followed for only one month; a longer follow-up period would help in better assessment of the diagnostic accuracy of various parameters.

## CONCLUSIONS

Current spirometry criteria may fail to identify some cases of bronchial asthma in clinically suspected patients. Utilizing *FEF2575* reversibility along with standard parameters increase diagnostic sensitivity and may aid in timely confirmation of asthma, particularly in patients who do not meet traditional *FEV1* or FVC reversibility thresholds, potentially improving clinical outcomes through more accurate diagnosis.
